# Baseline Susceptibility and Resistance Allele Frequency in *Ostrinia furnacalis* in Relation to Cry1Ab Toxins in China

**DOI:** 10.3390/toxins14040255

**Published:** 2022-04-02

**Authors:** Xiaobei Liu, Shen Liu, Ying Long, Yueqin Wang, Wenlu Zhao, Su Mon Shwe, Zhenying Wang, Kanglai He, Shuxiong Bai

**Affiliations:** 1 State Key Laboratory for the Biology of the Plant Diseases and Insect Pests, Institute of Plant Protection, Chinese Academy of Agricultural Sciences, Beijing 100193, China; liuxiaobei402@163.com (X.L.); liushen8152@163.com (S.L.); yueqinqueen@126.com (Y.W.); sumonshwe.ento@gmail.com (S.M.S.); wangzhenying@caas.cn (Z.W.); hekanglai@caas.cn (K.H.); 2 Syngenta Biotechnology (China) Co., Ltd., Zhongguancun Life Science Park, No. 25, Life Science Park Road, Changping District, Beijing 102206, China; ying.long@syngenta.com; 3 Dezhou Academy of Agricultural Sciences, Dezhou 253000, China; zhaowenludy@163.com

**Keywords:** *Ostrinia furnacalis*, susceptibility, resistance allele frequency, Cry1Ab

## Abstract

Asian corn borer (ACB), *Ostrinia furnacalis* (Guenée) is a destructive pest of corn and major target of transgenic corn expressing Bt (*Bacillus thuringiensis*) toxins in China. It is necessary to establish the baseline susceptibility of geographically distinct ACB populations to Cry1Ab protein and estimate the resistance alleles frequency. The median lethal concentration (LC_50_) and LC_95_ values of Bt toxin Cry1Ab for 25 geographically distinct populations collected in 2018–2019 ranged from 0.86 to 71.33, 18.58 to 5752.34 ng/cm^2^, respectively. The median effective concentration (EC_50_) and EC_95_ values ranged from 0.03 to 10.40 ng/cm^2^ and 3.75 to 172.86 ng/cm^2^, respectively. We used the F_2_ screening method for estimating the expected frequency of resistance alleles of the 13 ACB populations, to Bt corn (Bt11 × GA21) expressing the Cry1Ab toxin. The neonates could not survive on the leaves of transgenic maize Bt11 × GA21 with *cry1Ab* gene, the Cry1Ab resistance allele frequency was still low in each geographic population in the field, ranging from 0.0032–0.0048, indicating that the sensitivity of ACB to Cry1Ab was still at a high level, and there were no viable resistant individuals in the field at present. The susceptibility of 25 populations of ACB collected in China showed regional differences, although the Cry1Ab resistance allele frequency in these ACB populations is still at a low level. This provides essential knowledge for making the decision to commercialize Bt maize, and monitoring resistance development and evaluating resistance management strategies in the future in China.

## 1. Introduction

The Asian corn borer (ACB) (*Ostrinia furnacalis* (Guenée)) is a major pest of corn and is extensively widespread in corn-growing countries, particularly in Asia and Southeast Asia [1,2]. ACB can attack the corn plant from seedling until harvest time and reduce corn yield and grain quality; the larvae feed on leaves, stalks, and tassels and bore into cornstalks and ears [3,4]. In China, it remains the most significant economic insect pest of corn, causing annual losses ranging from 6 to 9 million tons; it causes about 10% grain yield loss annually and may exceed 30% loss during years with severe infestation [5,6]. Several control strategies have been reported to reduce ACB infestation: planting resistant varieties, cultural control, chemical insecticide application, planting genetically modified maize (Bt transgenic corn), and biological approaches (www.plantwise.org/knowledgebank/pmdg/20167800596, accessed on 1 March 2022). Chemical control is widely used to control ACB among all the options for ACB control. However, most farmers rely on pesticides which, among other problems, may lead to environmental contamination, risk to human health, non-target effects on natural enemies, and the development of resistance [7]. Controlling ACB is difficult because it lives inside its host plants during most of its life cycle [8,9,10].

With the development of new technology, *Bacillus thuringiensis* (Bt) transgenic corn can provide an effective method for the control of ACB and European corn borer (ECB), *O. nubilalis* (Hübner) [11,12,13]. Planting Bt transgenic corn controls the whole growth period of target insects [11], and it has advantages in reducing the amounts of chemical pesticide used, protecting the environment, and increasing economic benefits [14,15,16]. Bt transgenic corn has been commercially grown in the United States, Canada, Argentina, South Africa, and other countries, over an area of 1.70 million ha in 1996. Since then, the global planted area of Bt corn was 60.9 million ha in 2019, equivalent to 32% of the global planted area of corn [17]. However, the large-scale planting of transgenic Bt crops has an unintended consequence of targeting insects to undergo certain pressures, for example, Bt protein selection pressure, and after a long time, it leads to the generation of resistant populations of target insects. So far, *Helicoverpa zea* (Boddie) [18], *Spodoptera frugiperda* (Smith) [19,20], *Diabrotica virgifera virgifera* Leconte [21,22], *Diatraea saccharalis* (Fabricius) [23], *Striacosta albicosta* (Smith) [24,25], *O. nubilalis* [26], and *Busseola fusca* (Fuller) [27] have developed resistance to transgenic Bt plants. Therefore, to realize the sustainable application of Bt transgenic crops, it is necessary to implement reasonable resistance management strategies to avoid or delay the evolution of resistance of target insects. Moreover, it is of great significance for the sustainable application of transgenic crops to monitor the resistance of target insects and understand the evolution of resistance in the field population. 

Although Bt corn has not yet been commercially planted in China so far, in 2020–2021, several Bt corn events have been awarded a safety certificate by the Ministry of Agriculture and Rural Affairs of the People’s Republic of China, which means Bt corn will be planted in the country in the near future. Therefore, China has been pushing the pace on the commercial planting of Bt corn (http://www.moa.gov.cn/ztzl/zjyqwgz/spxx/202101/t20210111_6359740.htm, accessed on 1 March 2022). Existing evidence shows that Bt corn controls target pests throughout their growth period [11].

Before the commercial planting of transgenic Bt crops, it is crucial to establish baseline data on susceptibility and the frequency of resistance alleles of target insects to Bt proteins to allow resistance monitoring, and the formulation and implementation of the aggressive management strategies are also of great significance. The previous studies reported the baseline susceptibility of ACB to Cry1Ab protein in the major corn-growing regions [28], and resistance allele frequencies to Cry1Ab, Cry1Ac, and Cry1F in ACB populations collected from Huanghuaihai (River Valley) Summer Corn Region in China [29]. However, their collection range is relatively small, representing sensitivity of ACB to Bt in some areas of China, which has certain limitations or deficiencies for the sensitive detection of ACB in Bt corn before widespread commercial planting in China.

In this study, the baseline susceptibility and Cry1Ab resistance allele frequency of ACB populations were analyzed in most corn planting areas of China, which provide a theoretical basis for the study of transgenic insect-resistant maize and the development of ACB resistance monitoring and resistance management strategies in China.

## 2. Results

### 2.1. Baseline Susceptibility of ACB to Bt Cry1Ab Protein

The toxicity of Cry1Ab insecticidal protein to different geographical populations of ACB is shown in Table 1. LC_50_ and LC_95_ values ranged from 0.86 ng/cm^2^ (Dandong) to 71.33 ng/cm^2^ (Luoyang) and from 18.58 ng/cm^2^ (Qinhuangdao) to 5752.34 ng/cm^2^ (Luoyang), respectively. There were significant differences (*p* < 0.05) in susceptibility among the populations tested; for example, the Dandong population had the highest sensitivity to Cry1Ab protein. The difference between the most sensitive and least sensitive populations was 82.9 times at the LC_50_ level. The LC_50_ values of 10 geographic populations were significantly higher than those of the laboratory population, most of which were from the HuangHuaiHai Summer Corn Region and the Northeast Spring Corn Region. The LC_50_ of Cry1Ab protein demonstrated regional differences among different geographical populations. The LC_50_ of Cry1Ab protein was higher in the HuangHuaiHai Summer Corn Region, such as Luoyang and Xinxiang of Henan Province, Xiaoxian of Anhui Province, and Dezhou of Shandong Province, and so forth.

### 2.2. ACB Larval Growth Inhibition to Bt Cry1Ab Protein

The larval growth inhibition of different ACB geographical populations to Cry1Ab insecticidal protein are presented in Table 2. EC_50_ and EC_95_ values ranged from 0.03 ng/cm^2^ (Shenyang) to 10.40 ng/cm^2^ (Xinxiang) and from 3.75 ng/cm^2^ (Qinhuangdao) to 172.86 ng/cm^2^ (Xinxiang), respectively. Among the 25 measured populations, the Shenyang population had the highest sensitivity to Cry1Ab protein, in contrast to the Xinxiang population, which had the lowest sensitivity. The difference between the most sensitivity and least sensitivity populations was 346.7 times at the EC_50_ level. The EC_50_ values of six geographic populations were significantly higher than those of the laboratory population. Most of these populations were collected from the HuangHuaiHai Summer Corn Region and the Northeast Spring Corn Region.

### 2.3. Resistance Allele Frequencies Related to Cry1Ab

Neonates of all the 13 tested ACB populations did not survive on the leaves of transgenic maize Bt11 × GA21 expressing Cry1Ab toxin. The results showed that the frequency of Cry1Ab resistance allele was still low in each geographic population in the field, indicating that the sensitivity of ACB to Cry1Ab was still at a high level, and there were no viable resistant individuals in the field at present (Table 3).

## 3. Discussion

Long-term large-scale planting of Bt corn may lead to the resistance of target pests to Bt protein, potentially negating the insecticidal effect of Bt corn and the application of other Bt crops. Therefore, it is vital to understand the baseline susceptibility and initial resistance allele frequencies of the ACB population to Bt protein before the widespread commercialization of Bt corn in China. A total of 25 ACB populations were collected in 2018–2019 from distinct geographic populations to understand the variations in their susceptibility to Bt protein Cry1Ab and estimate the Cry1Ab resistance allele frequency. Our study provides data support for monitoring the resistance of the ACB population to Cry1Ab protein.

The results from our study demonstrated that Cry1Ab protein is highly toxic to ACB populations in China, which concurs with the results of He et al. (2005) and Li et al. (2020). Additionally, ACB populations in Vietnam and the Philippines were highly susceptible to Cry1Ab protein [30]. We also found that the susceptibility of distinct ACB geographical populations to Cry1Ab protein showed some regional differences. However, interpopulation variation in susceptibility to Bt has been reported in corn borers. Stone and Sims (1993) found that the susceptibility to Cry1Ac protein of the American populations of *H. zea* and *Heliothis virescens* varied considerably between populations [31]. There are also significant interpopulation variations in susceptibility to Cry1Ab and Cry1Ac protein in the ECB, which are thought to reflect natural variation in Bt susceptibility between populations rather than changes due to prior selection pressures [32]. He et al. (2005) and Li et al. (2020) verified that the susceptibilities of different ACB populations to Cry1Ab protein were significantly different among different populations in China [28,29]. In a similar study, geographical variations in susceptibility to Cry1Ab protein were observed in 11 populations of ACB in Vietnam, which may be due to the natural variability between populations [33]. However, no studies have shown that variations in susceptibility are influenced by pheromone race, voltine ecotype, or geographic location.

The regional differences in susceptibility to Cry1Ab protein among different ACB geographical populations were significant, and the range of variation at the LC_50_ level was less than 83-fold. Bt formulations as an effective biological control method have been used for corn pest control in the 1980s by spraying or delivering granules by airplane [34]. However, because of the high cost and the lack of technology of Bt formulation, farmers have seldom used Bt formulation to control pests in the last decade [6]. These studies suggest that regional differences in susceptibility of ACB populations to Cry1Ab protein reflect natural variation in Bt susceptibility among the distinct geographical populations.

This study demonstrated that the susceptibility of different ACB populations to Cry1Ab protein has considerable inter-population variation, which may be related to the corn-growing areas and crop types in China. The susceptibility of target pests to Bt protein is different due to different maize planting areas, crop types and proportions, ecological environment, and other factors [35,36]. In addition, the method of protein preparation, the activation of trypsin, purification method, and protein quantification will affect the virulence of Bt protein on target pests [37]. The feeding of ACB field populations on transgenic cotton may indirectly contribute to the increased resistance of ACB to Cry1Ab protein in the Huang-Huai-Hai Summer Corn Region.

The F_2_ generation detection method has the characteristic of having an ability to detect recessive resistant individuals with higher sensitivity [38], which has been widely used in monitoring the resistance alleles of *O. nubilalis* [39], *Plutella xylostella* (L.) [40] and *Tryporyza incertulas* (Walker) [41]. Li et al. (2020) used diagnostic concentration (93 ng/cm^2^) to estimate the resistance allele frequency of Cry1Ab to the ACB population of Huang-Huai-Hai Summer Corn Region, China. They reported that the initial frequency of resistance alleles was 0.002 [29]. We did not find alleles in all 13 ACB populations conferring resistance to Bt corn, which express the Cry1Ab toxin. These results reflect an assessment that the frequency of resistant alleles in ACB population in China is still low. Although Bt corn has not yet been commercially planted in China so far, in 2020, several Bt corn events (“DBN9936” and “Ruifeng125”) have been awarded a safety certificate by the Ministry of Agriculture and Rural Affairs of the People’s Republic of China, which means Bt corn will be planted in the country in the near future. Therefore, China has been pushing the pace on the commercial planting of Bt corn (http://www.moa.gov.cn/ztzl/zjyqwgz/spxx/202101/t20210111_6359740.htm, accessed on 1 March 2022). Thus, our results, by providing baseline data on allele frequency of resistant genes and susceptibility of ACB to Bt toxin, lay a foundation for resistance detection of transgenic corn after the widespread planting in China that will occur in the near future. 

## 4. Conclusions

We report here on the baseline susceptibility of distinct geographical populations of ACB to Cry1Ab toxin, which is crucial for establishing an effective ACB resistance monitoring program in China. Our findings collectively pointed that ACB populations in China have high susceptibility to Cry1Ab currently, though there are also regional differences in toxin sensitivity. The Cry1Ab resistance allele frequency in the tested geographical ACB populations is still at a low level. The study will lay the foundation for resistance detection of transgenic maize after commercial planting in China.

## 5. Materials and Methods

### 5.1. Field Collections

**Figure 1:**
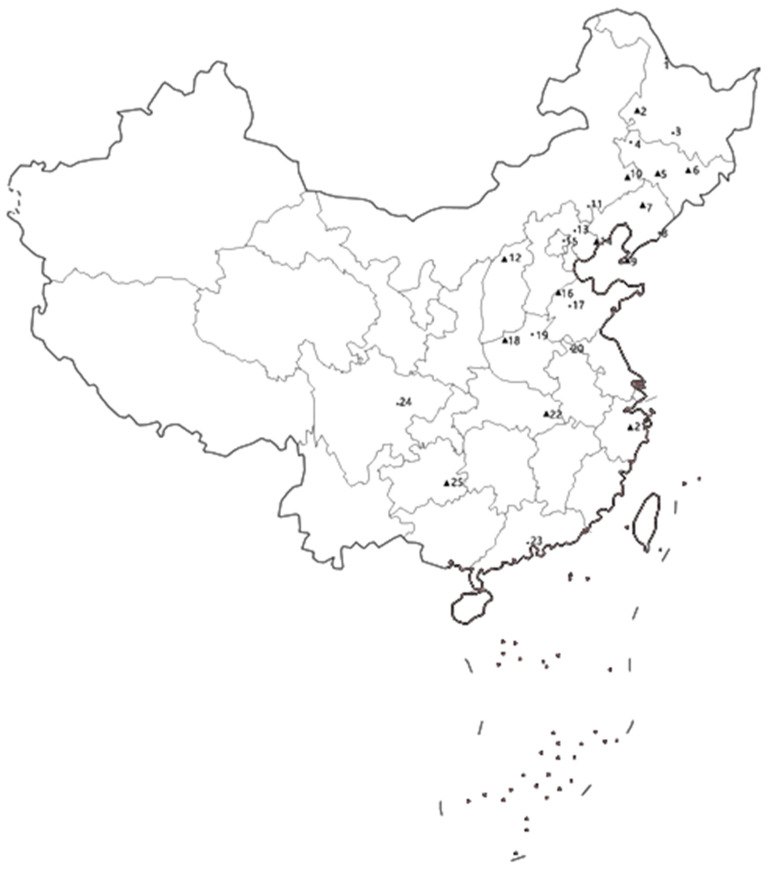
Sampling locations where *O. furnacalis* were collected. “•” means only baseline was done; “▲” means baseline and resistance allele frequency were performed. The numbers on the map correspond to the location mentioned in Table 4.

A total of 25 ACB field populations were collected in cornfields from different geographic regions of 15 provinces in China, Heilongjiang, Liaoning, Jilin, Inner Mongolia, Shanxi, Hebei, Beijing, Henan, Hubei, Anhui, Shandong, Zhejiang, Sichuan, Guizhou, Guangdong, from 2018 to 2019 (Figure 1). See Table 4 for detailed information.

### 5.2. Susceptible Population

A lab susceptible population (collected in Shaanxi province in 2000) reared on an artificial diet without exposure to any Bt proteins for several generations was used as a reference. These larvae were reared to pupae on a maize flour–wheatgerm–agar diet [42]. All the ACB populations were reared at 27–28 °C and 70–80% relative humidity (RH) under a photoperiod of 16:8 h (L:D).

### 5.3. Cry1Ab Protein

Trypsin-activated Cry1Ab insecticidal protein (powder type) was provided by Syngenta Biotechnology Co., Ltd., Beijing, China. with 84.3% of purity and stored at −80 °C. Afterward, the protein was solubilized in 10 mM ammonium bicarbonate (pH 10.0) with 1 mM DTT. Serial dilutions of seven or twelve solutions were made to decrease concentrations of the Cry1Ab protein were made with 10 mM ammonium bicarbonate (pH 10.0), then treated with surfactant 0.1% Triton X-100 to obtain a uniform coverage on the diet surface.

### 5.4. Bt Corn

Corn hybrid Bt11 × GA21 and a closely related non-Bt corn hybrid were raised by Syngenta, planted in a greenhouse in Beijing. GM corn Bt11 × GA21 express novel proteins Cry1Ab and mEPSPS. Bt11 expresses the insecticidal protein Cry1Ab which confers resistance to lepidopteran insect pests while the *mepsps* gene in GA21 confers tolerance to broad spectrum non-selective glyphosate herbicide.

### 5.5. Baseline Bioassay

F_1_ neonates from different populations (minimum 30 female moths) were pooled and used for concentration-response bioassays using the diet-overlay method. Approximately 1 mL of diet (Same as the diet of lab-susceptible populations rearing) was dispensed into each well of a 24-well plate and allowed to solidify, and then 50 µL of the formulated solution or control was overlaid on the diet using a pipette to produce uniform coverage of the Bt toxin on the diet surface. After drying, neonate larvae (<24 h) were released in each well with a 2 cm^2^ area by using a fine brush. The plates were sealed with a membrane (Cat# 3 M-9733, Minnesota Mining and Manufacturing Company, Saint Paul, Minnesota, USA) perforated with a sharp pin on each cell to provide aeration. Plates were maintained in the rearing room at 28 ± 1 °C, 60 ± 10% relative humidity, and a 14:10 h = L:D. About 1728 or 2808 neonates in total for each geographical population bioassay and each concentration with three replicates (*n* = 216), eight or thirteen Cry1Ab concentrations (including control) were used for each dose of Cry1Ab proteins, and a buffer solution treated with Triton X-100 was used as a control. Larval mortality and the weight of surviving larvae were recorded after seven days of inoculation. Larvae that do not pass the first instar were considered dead.

### 5.6. Resistance Allele Frequency

The female moths collected (F_0_) were reared separately, and only egg masses from a female moth with one spermatheca (indicating the female mate only once) were recognized as the offspring of a family (F_1_).

Collected larvae (F_0_) were reared up to the pupal stage on a non-Bt artificial diet. All similarly aged pupae were sexed and paired. Each couple was placed in individual oviposition containers (500 mL cups) for mating to establish a family (F_1_). Collected pupae (F_0_) were treated the same as the pupal stage of collected larvae.

Each family was maintained on a non-Bt artificial diet, which was sib-mated to produce F_2_ larvae, which were used for the frequency of Cry1Ab resistance allele studies (minimum 50 families for each geographic population) by leaf disc bioassay as described below.

F_1_ mass-mated adults produced F_2_ larvae, which were split into two groups. From the first group, 48 F_2_ larvae from each family were screened on Bt11 × GA21 leaf tissues. During the V6-V9 growth stages of Bt11 × GA21, the middle parts of whorl leaves were excised from the corn plants grown in the greenhouse and cut into squares (1 cm × 1 cm) and then placed into a 24-well plate with 1% agar (500 μL per well). The second group (48 F_2_ larvae) was fed on non-BT maize leaf tissue according to different families. There were two 24-well plates per treatment (Bt and non-Bt) for each family. Each well of the plates was released with a single neonate. Within the observation period, leaf discs were regularly replaced as needed. The number of surviving larvae and larval mortality were recorded on the 7th day.

Surviving larvae were (F_2_) screened using Bt11 × GA21 leaf tissues as an indicator that their parents may potentially carry a Cry1Ab resistance allele. Hence, the corresponding F_2_ larvae on non-Bt corn plants were identified and carried forward to F_3_. A confirmatory test was performed at F_3_ for only families that show larval survivorship during the F_2_ screening. The F_2_ adults from the non-Bt corn plants (control) were mass mated to generate the following generation to test on Bt leaf tissues, similar to the F_2_ screening above. Based on the number of survival larvae in F_2_, the Cry1Ab resistance allele frequency for each population was calculated following Andow and Alstad, 1998 [38].

### 5.7. Statistical Analysis

The susceptibility of different populations to Cry1Ab was analyzed by probit regression using PoloPlus (LeOra Software, Version 1.0), which would generate LC_50_ values with 95% fiducial limits (FL), Chi-Square (*χ*^2^), slope with standard errors (Slope ± SE), and degrees of freedom (*df*). The difference between LC_50_ values and LC_95_ values in diet-overlay bioassays was considered significant differences if their 95% fiducial limits did not overlap.

The estimates of EC_50_ and EC_95_, their respective confidence intervals, and the weight of the surviving larvae at each concentration were evaluated by analyzed by non-linear regression. Statistical data were analyzed by SAS 9.2.

The formula for the calculation of frequency of resistance allele and relevant variances were as follows according to Andow and Alstad, 1998 [38]:

$$E\left(q\right)=\frac{s+1}{4\left(n+2\right)}$$

$$\mathrm{Var}\left(q\right)=\;E\left(q\right)\frac{1-E\left(q\right)}{n+3}$$

$$95\%\;\mathrm{CI}\;=\;E\left(q\right)\;\pm \;\left(1.96\times \sqrt{\mathrm{Var}\left(q\right)}\right)$$

E(*q*): the expected frequency of resistance allele.

Var(*q*): the variance associated with the expected frequency.

*s*: the number of female families with resistance allele.

*n*: the number of female families tested.

## Figures and Tables

**Table 1:** Baseline susceptibility of different Chinese ACB populations to Cry1Ab protein.

Population	N	LC_50_ (95% FL) ng/cm^2 a^	LC_95_ (95% FL) ng/cm^2 a^	Slope ± SE	*χ* ^2^	*df* (*χ*^2^)
Luoyang	1512	71.33 (45.61–102.64) a	5752.34 (2532.54–21,631.84) a	0.86 ± 0.11	10.30	19
Xinxiang	1512	66.31 (46.99–90.11) a	3708.90 (1901.84–9857.31) a	0.94 ± 0.10	8.78	19
Xiaoxian	1512	55.18 (35.31–80.01) a	4596.70 (2166.61–14,375.89) a	0.86 ± 0.10	11.51	19
Dezhou	1512	47.96 (35.01–61.51) a	1052.27 (679.00–1981.37) b	1.23 ± 0.13	7.73	19
Jinan	2160	28.35 (22.50–34.42) b	305.75 (246.59–397.45) cd	1.59 ± 0.11	8.56	28
Qiqihar	1512	27.86 (20.05–36.66) b	922.94 (603.31–1625.22) b	1.08 ± 0.09	7.77	19
Gongzhuling	1944	25.56 (20.23–30.95) b	236.60 (190.22–311.10) cd	1.70 ± 0.13	10.34	25
Harbin	1296	23.92 (7.76–31.17) bc	1079.23 (684.77–1941.41) b	0.99 ± 0.07	5.95	16
Dongyang	1944	13.67 (10.11–17.41) c	183.25 (146.29–241.62) d	1.46 ± 0.11	11.14	25
Huanggang	1728	13.60 (10.51–16.73) c	138.11 (110.60–182.76) de	1.63 ± 0.13	10.72	22
Dalian	1944	10.40 (7.54–13.60) cd	229.70 (178.06–310.82) cd	1.22 ± 0.08	6.57	25
Heihe	1512	8.92 (7.12–10.85) d	109.66 (87.64–143.22) e	1.51 ± 0.09	9.10	19
Miyun	1728	7.94 (5.83–10.26) de	143.27 (111.27–194.29) de	1.31 ± 0.09	10.46	22
Kaili	1728	7.11 (5.14–9.30) de	145.37 (111.80–199.33) de	1.26 ± 0.09	12.41	22
Yingxian	1512	6.98 (4.67–9.65) de	1003.45 (567.19–2173.66) b	0.76 ± 0.06	3.77	19
Tongliao	1512	6.69 (4.59–8.76) de	53.76 (43.45–71.26) f	1.82 ± 0.19	12.01	19
Guangzhou	1728	5.75 (4.59–6.98) e	63.81 (51.90–81.29) f	1.57 ± 0.09	6.76	22
Chifeng	1944	5.20 (3.54–7.18) ef	250.65 (182.58–363.95) cd	0.98 ± 0.06	13.13	25
Dunhua	1512	4.67 (3.55–5.87) ef	84.38 (65.58–114.83) ef	1.31 ± 0.09	11.29	19
Deyang	1944	3.90 (2.89–5.06) f	109.87 (84.15–149.65) e	1.14 ± 0.06	4.19	25
Baicheng	1728	2.97 (2.15–3.91) f	99.81 (74.12–142.10) e	1.08 ± 0.07	10.42	22
Qinhuangdao	1296	1.88 (1.39–2.40) g	18.58 (14.74–24.64) h	1.65 ± 0.13	12.72	16
Shenyang	2016	1.69 (1.13–2.31) g	62.98 (48.62–86.72) f	1.05 ± 0.08	10.41	26
Chengde	1512	1.36 (1.00–1.75) gh	22.40 (17.39–30.43) gh	1.35 ± 0.09	10.15	19
Dandong	1512	0.86 (0.44–1.38) h	33.87 (25.47–49.09) g	1.03 ± 0.10	7.09	19
Laboratory population	360	6.28 (4.46–8.50) de	57.33 (38.35–98.88) fg	1.71 ± 0.17	10.40	13

N = Number of larvae in the probit analysis. 95% FL = 95% fiducial limits. ^a^ = Values followed by the same lowercase letter in the same column indicate no significant difference (overlapping 95% fiducial limits). SE = Standard error. *χ*^2^ = Chi-square. *df* (*χ*^2^) = Degrees of freedom.

**Table 2:** The larval growth inhibition of Cry1Ab protein to different ACB geographical populations.

Population	N	EC_50_ (95% FL) ng/cm^2 a^	EC_95_ (95% FL) ng/cm^2 a^	Slope ± SE	*χ* ^2^	*df* (*χ*^2^)
Xinxiang	1512	10.40 (9.03–11.85) a	172.86 (135.65–231.00) a	1.35 ± 0.07	20.10	19
Heihe	1512	7.09 (6.14–8.09) b	45.09 (36.42–58.99) cd	2.05 ± 0.13	29.25	19
Xiaoxian	1512	4.56 (3.73–5.41) c	77.17 (61.58–101.50) bc	1.34 ± 0.08	23.03	19
Dezhou	1512	3.45 (2.65–4.30) c	95.58 (73.09–133.61) b	1.14 ± 0.08	7.05	19
Qiqihar	1512	3.30 (2.61–4.00) c	55.24 (44.23–72.58) c	1.34 ± 0.09	22.86	19
Chifeng	1944	1.68 (1.35–2.03) d	45.73 (35.81–61.17) cd	1.15 ± 0.06	19.61	25
Dunhua	1512	1.32 (0.86–1.80) de	18.95 (15.41–24.65) e	1.42 ± 0.13	15.44	19
Harbin	1296	1.25 (0.78–1.76) de	34.71 (26.43–49.49) d	1.14 ± 0.10	18.87	16
Yingxian	1512	1.23 (0.75–1.78) de	49.26 (37.38–70.47) cd	1.03 ± 0.09	6.80	19
Luoyang	1512	0.77 (0.38–1.27) e	65.46 (47.00–102.33) bc	0.85 ± 0.08	4.15	19
Miyun	1728	0.74 (0.56–0.93) e	16.82 (13.26–22.53) ef	1.21 ± 0.08	14.53	22
Gongzhuling	1944	0.68 (0.48–0.91) e	26.28 (20.23–36.17) de	1.04 ± 0.07	6.46	25
Dalian	1944	0.64 (0.46–0.84) e	15.81 (12.46–21.18) ef	1.18 ± 0.08	10.38	25
Baicheng	1728	0.64 (0.46–0.84) e	15.10 (11.87–20.34) ef	1.20 ± 0.09	4.07	22
Tongliao	1512	0.60 (0.43–0.77) e	7.83 (6.33–10.26) g	1.47 ± 0.12	4.58	19
Qinhuangdao	1296	0.60 (0.45–0.75) e	3.75 (3.15–4.73) h	2.08 ± 0.21	7.77	16
Kaili	1728	0.59 (0.40–0.79) ef	18.12 (14.04–24.88) ef	1.10 ± 0.08	3.61	22
Dongyang	1944	0.55 (0.38–0.74) ef	12.62 (9.96–16.97) f	1.21 ± 0.09	2.47	25
Deyang	1944	0.51 (0.35–0.68) ef	9.84 (7.84–13.08) fg	1.28 ± 0.10	11.24	25
Huanggang	1728	0.47 (0.30–0.67) ef	20.19 (15.38–28.39) e	1.01 ± 0.08	3.65	22
Chengde	1512	0.44 (0.29–0.59) ef	4.17 (3.43–5.40) h	1.68 ± 0.17	2.66	19
Guangzhou	1728	0.34 (0.20–0.49) f	8.36 (6.57–11.34) g	1.18 ± 0.11	3.78	22
Jinan	2160	0.20 (0.10–0.34) f	34.56 (24.98–51.65) d	0.74 ± 0.06	6.12	28
Dandong	1512	0.04 (0.00–0.20) g	4.41 (2.13–6.92) gh	0.81 ± 0.17	2.23	19
Shenyang	2016	0.03 (0.00–0.13) g	3.87 (1.89–6.01) h	0.76 ± 0.14	1.47	26
Laboratory population	360	0.46 (0.06–1.06) ef	12.21 (7.35–33.76) fg	1.16 ± 0.27	4.26	13

N = Number of larvae in the probit analysis. 95% FL = 95% fiducial limits. ^a^ = Values followed by the same lowercase letter in the same column indicate no significant difference (overlapping 95% fiducial limits). SE = Standard error. *χ*^2^ = Chi-square. *df* (*χ*^2^) = Degrees of freedom.

**Table 3:** The frequency of Cry1Ab resistance allele of different ACB geographical populations.

Province	Location	Number of Isofemale Lines	Var (*q*)	E(*q*) (95% CI)	Number of Isofemale Lines with Resistance
Jilin	Dunhua	61	6.18 × 10^−5^	0.0040 (0–1.95 × 10^−2^)	0
Liaoning	Shenyang	56	7.27 × 10^−5^	0.0043 (0–2.10 × 10^−2^)	0
Heilongjiang	Qiqihar	74	4.26 × 10^−5^	0.0033 (0–1.60 × 10^−2^)	0
Henan	Luoyang	60	6.38 × 10^−5^	0.0040 (0–1.97 × 10^−2^)	0
Shanxi	Yingxian	50	9.03 × 10^−5^	0.0048 (0–2.34 × 10^−2^)	0
Shandong	Dezhou	55	7.53 × 10^−5^	0.0044 (0–2.15 × 10^−2^)	0
Jilin	Gongzhuling	52	8.38 × 10^−5^	0.0046 (0–2.26 × 10^−2^)	0
Liaoning	Dalian	75	4.15 × 10^−5^	0.0032 (0–1.57 × 10^−2^)	0
Inner Mongolia	Tongliao	68	5.01 × 10^−5^	0.0036 (0–1.75 × 10^−2^)	0
Hebei	Qinhuangdao	74	4.26 × 10^−5^	0.0033 (0–1.60 × 10^−2^)	0
Hubei	Huanggang	58	6.80 × 10^−5^	0.0042 (0–2.03 × 10^−2^)	0
Zhejiang	Dongyang	66	5.31 × 10^−5^	0.0037 (0–1.80 × 10^−2^)	0
Guizhou	Kaili	53	8.08 × 10^−5^	0.0045 (0–1.95 × 10^−2^)	0
Total	-	802	3.86 × 10^−7^	0.0003 (0–1.52 × 10^−3^)	0

**Table 4:** Description of different ACB geographical populations in 2018–2019.

Province	Location	Coordinates	Corn Ecological Region	Collected Insect Life Stage	Collection Month	Number of Collected Insects
Heilongjiang	Heihe (1)	50°14′58.51″ N; 127°29′56.48″ E	NESpC	female moths	July, 2018	70
Heilongjiang	Qiqihar (2)	47°20′53.08″ N; 123°57′12.55″ E	NESpC	female moths	July, 2018	184
Heilongjiang	Harbin (3)	45°45′25.08″ N; 126°38′32.87″ E	NESpC	female moths	July, 2018	85
Jilin	Baicheng (4)	45°37′8.49″ N; 122°50′28.01″ E	NESpC	female moths	July, 2019	152
Jilin	Gongzhuling (5)	43°30′16.31″ N; 124°49′21.58″ E	NESpC	female moths	July, 2019	120
Jilin	Dunhua (6)	43°22′22.94″ N; 128°13′56.71″ E	NESpC	female moths	July, 2018	160
Liaoning	Shenyang (7)	41°47′48.36″ N; 123°25′44.75″ E	NESpC	female moths	June, 2018	195
Liaoning	Dandong (8)	40°7′27.47″ N; 124°22′58.96″ E	NESpC	female moths	July, 2018	203
Liaoning	Dalian (9)	38°54′52.52″ N; 121°37′7.04″ E	NESpC	female moths	June, 2019	180
Inner Mongolia	Tongliao (10)	43°37′2.74″ N; 122°15′47.23″ E	NESpC	female moths	June, 2019	169
Inner Mongolia	Chifeng (11)	42°16′31.14″ N; 118°57′24.50″ E	NESpC	female moths	June, 2019	220
Shanxi	Yingxian (12)	39°33′10.04″ N; 113°11′25.87″ E	NSpC	female moths	August, 2018	201
Hebei	Chengde (13)	40°58′34.33″ N; 117°56′20.95″ E	HHHSuC	female moths	June, 2019	117
Hebei	Qinhuangdao (14)	39°56′33.11″ N; 119°35′11.68″ E	HHHSuC	female moths	June, 2019	143
Beijing	Miyun (15)	40°22′34.25″ N; 116°50′34.62″ E	HHHSuC	female moths	August, 2019	132
Shandong	Dezhou (16)	37°27′14.28″ N; 116°18′26.74″ E	HHHSuC	female moths	August, 2018	174
Shandong	Jinan (17)	36°40′32.91″ N; 117°0′3.32″ E	HHHSuC	female moths	August, 2019	75
Henan	Luoyang (18)	34°39′46.95″ N; 112°26′4.08″ E	HHHSuC	female moths	August, 2018	186
Henan	Xinxiang (19)	35°18′9.42″ N; 113°53′2.37″ E	HHHSuC	female moths	August, 2018	130
Anhui	Xiaoxian (20)	34°11′16.44″ N; 116°56′43.66″ E	HHHSuC	female moths	September, 2018	182
Zhejiang	Dongyang (21)	29°17′21.91″ N; 120°14′30.66″ E	SEHiC	pupae	July, 2019	210
Hubei	Huanggang (22)	30°26′51.76″ N; 114°52′45.71″ E	SEHiC	female moths	August, 2019	266
Guangdong	Guangzhou (23)	23°7′30.64″ N; 113°16′50.29″ E	SEHiC	larvae	August, 2019	143
Sichuan	Deyang (24)	31°7′40.77″ N; 104°23′55.14″ E	SWHiC	larvae	September, 2019	172
Guizhou	Kaili (25)	26°34′0.80″ N; 107°58′52.75″ E	SWHiC	larvae	August, 2019	225

NESpC, Northeast Spring Corn Region; NSpC, North Spring Corn Region; HHHSuC, Huanghuaihai (River Valley) Summer Corn Region; SEHiC, Southeast Hilly Corn Region; SWHiC, Southwest Hilly Corn Regio.

## Data Availability

Not applicable.
